# First person – Trace Stay

**DOI:** 10.1242/dmm.043356

**Published:** 2019-12-09

## Abstract

First Person is a series of interviews with the first authors of a selection of papers published in Disease Models & Mechanisms, helping early-career researchers promote themselves alongside their papers. Trace Stay is first author on ‘*In vivo* cerebellar circuit function is disrupted in an *mdx* mouse model of Duchenne muscular dystrophy’, published in DMM. Trace conducted the research described in this article while a PhD student in Roy V. Sillitoe's lab at the Baylor College of Medicine, Houston, TX, USA. He is now a postdoctoral scholar in the lab of Jennifer L. Raymond at Stanford University, Stanford, CA, USA, investigating how the cerebellum interacts with sensory input to influence motor control and more cognitive processing.


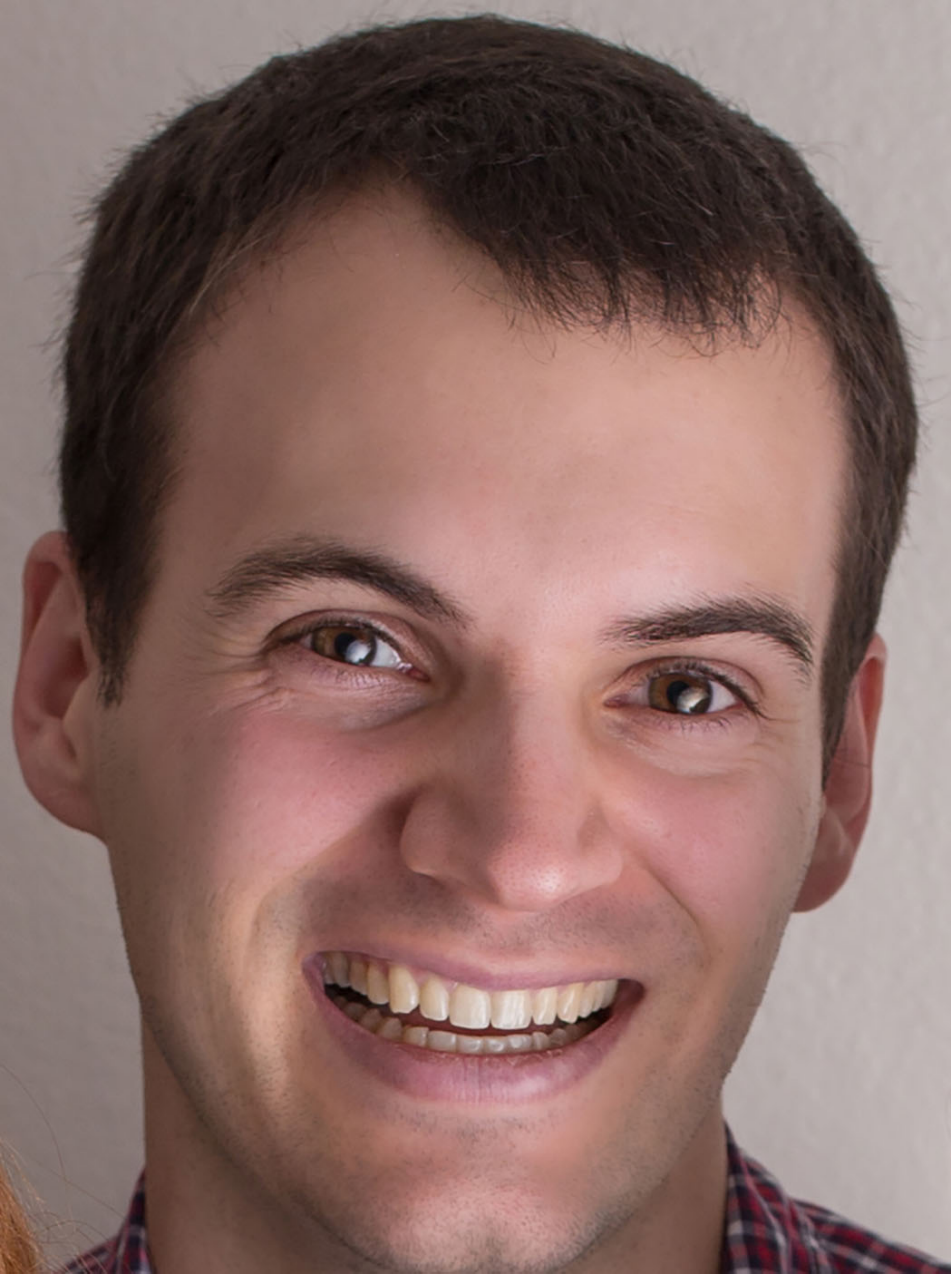


**Trace Stay**

**How would you explain the main findings of your paper to non-scientific family and friends?**

In the 1960s to 1970s, studies began suggesting that individuals with Duchenne muscular dystrophy (DMD) could have some degree of cognitive impairment along with the more obvious muscular deficits. In the years since, this finding has been linked to the loss of dystrophin protein that occurs in DMD, as dystrophin is expressed throughout the body, including in the brain. More recent research has shown that, in animal models of DMD, *in vitro* neural activity in the cerebellum is often abnormal. Since the cerebellum is heavily involved in both motor control and some non-motor tasks, these previous results indicated a possible explanation for some of the cognitive effects. However, no one had previously studied how the cerebellum functions in an intact, living brain in an animal model of DMD. Our paper establishes that, in *mdx* mice, the key integration cells and output cells of the cerebellum have significant changes in firing properties, which are not the result of cell death or anatomical restructuring, similar to what is seen in other disease models. This supports the hypothesis that the cerebellum could be a potential target for clinical treatment of the cognitive symptoms seen in individuals with DMD.

**What are the potential implications of these results for your field of research?**

Our results indicate the cerebellum is an important element to analyze when studying brain function in confirmatory studies of DMD in other animal species, such as dogs, pigs or rabbits. Ongoing research is continually uncovering cerebellar interactions with other brain regions, so it is likely that cerebellar changes could influence a wide variety of behaviors. Translational studies on mice models of dystonia and tremor are currently seeing remarkable beneficial effects utilizing experimental deep-brain stimulation in the cerebellar nuclei, and it is possible that similar approaches to neuromodulation could provide therapeutic effects in DMD.

“The fact that we see *in vivo* cerebellar deficits in *mdx* mice suggests there could be even more significant changes in humans, where muscles are severely affected.”

**What are the main advantages and drawbacks of the model system you have used as it relates to the disease you are investigating?**

The *mdx* mouse model of DMD has been heavily studied for three decades. As such, it is well known that, although the standard *mdx* mice lack full-length dystrophin, they do not show the same muscular symptoms or lifespan effects as seen in human DMD. This is partially due to an upregulation of utrophin and integrin to compensate for dystrophin loss in *mdx* mice, which is not seen in humans. However, this relative sparing of muscle is actually an advantage for our study, as is helps dissociate our observed cerebellar deficits from other compensatory functional changes that are known to occur when muscles deteriorate in other diseases. The fact that we see *in vivo* cerebellar deficits in *mdx* mice suggests there could be even more significant changes in humans, where muscles are severely affected.

**What has surprised you the most while conducting your research?**

It surprised me how early the cognitive effects in DMD were reported. Duchenne noted some degree of cognitive impairment in his five patients back in 1868. However, because they weren't the dominant symptoms and there was a lot of heterogeneity across individuals, the cognitive effects were under-reported for many decades after. It reminded me of non-motor symptoms detailed in individuals with cerebellar damage, which were initially described 100 years ago and which have been debated over ever since.

**Figure:**
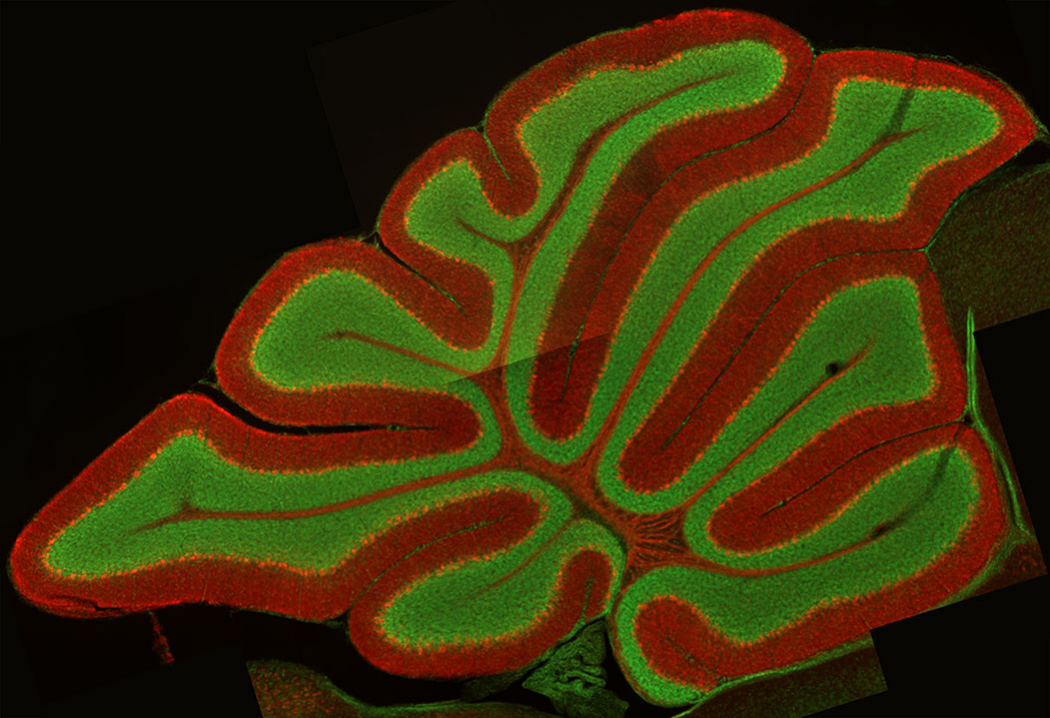
**The key cerebellar integrator Purkinje cells divide the cerebellum into an outer molecular layer and an inner granular layer (red, Purkinje cell somata, dendrites and inner axons stained with calbindin; green, granule cells marked with a Nissl stain).**

**Describe what you think is the most significant challenge impacting your research at this time and how will this be addressed over the next 10 years?**

In studying neural physiology, I think the biggest challenge over the next 10 years will be appropriate data analysis. Hardware has improved exponentially to where we can record hundreds or thousands of neurons relative to our independent variables of interest, so the issue now is how to identify the most significant effects. Questions like the effects of synchrony, attentional state and region-to-region oscillations are becoming salient, where before we didn't have the tools to adequately answer them. I know I've certainly been grateful to learn from other data scientists, which is the training I think that will become more and more indispensable.

**What's next for you?**

I am currently conducting postdoctoral research in the Raymond lab at Stanford studying the role of the cerebellum in oculomotor vestibular processing (and hopefully its dysfunction). My goal is to clarify cerebellar interactions with visual and motor feedback pathways, to better understand the basic biology that could eventually lead to vestibular therapeutics.

“[…] learning biology gives very practical knowledge that has real power in helping us make informed perceptions and decisions about our own health and our interactions with the natural world.”

**How would you explain why your research matters to the general public?**

Research is valuable on many different levels. For individuals, learning biology gives very practical knowledge that has real power in helping us make informed perceptions and decisions about our own health and our interactions with the natural world. Neuroscience in particular helps us understand mental health and cognitive disabilities in a completely different way than previous generations. Of course, basic research is also the foundation for clinical applications that improve human health, as well as public health policy that affects millions. Research on DMD could help hundreds of thousands of individuals who currently live with a debilitating and lethal condition.
